# BCGΔBCG1419c increased memory CD8^+^ T cell-associated immunogenicity and mitigated pulmonary inflammation compared with BCG in a model of chronic tuberculosis

**DOI:** 10.1038/s41598-022-20017-w

**Published:** 2022-09-22

**Authors:** Kee Woong Kwon, Michel de Jesús Aceves-Sánchez, Cristian Alfredo Segura-Cerda, Eunsol Choi, Helle Bielefeldt-Ohmann, Sung Jae Shin, Mario Alberto Flores-Valdez

**Affiliations:** 1 Department of Microbiology, Graduate School of Medical Science, Brain Korea 21 Project, Yonsei University College of Medicine, Seoul, 03722 South Korea; 2 Biotecnología Médica y Farmacéutica, Centro de Investigación y Asistencia en Tecnología y Diseño del Estado de Jalisco, A.C., Av. Normalistas No. 800, Col. Colinas de la Normal, 44270 Guadalajara, JAL Mexico; 3 Australian Infectious Diseases Research Centre, The University of Queensland, Saint Lucia, QLD Australia; 4 School of Chemistry and Molecular Biosciences, University of Queensland St. Lucia Campus, St Lucia, QLD 4072 Australia; 5 Institute for Immunology and Immunological Disease, Yonsei University College of Medicine, Seoul, 03722 South Korea

**Keywords:** Vaccines, Live attenuated vaccines, Pathogens, Cellular microbiology

## Abstract

Previously, we reported that a hygromycin resistant version of the BCGΔBCG1419c vaccine candidate reduced tuberculosis (TB) disease in BALB/c, C57BL/6, and B6D2F1 mice infected with *Mycobacterium tuberculosis* (Mtb) H37Rv. Here, the second-generation version of BCGΔBCG1419c (based on BCG Pasteur ATCC 35734, without antibiotic resistance markers, and a complete deletion of *BCG1419c*) was compared to its parental BCG for immunogenicity and protective efficacy against the Mtb clinical isolate M2 in C57BL/6 mice. Both BCG and BCGΔBCG1419c induced production of IFN-γ, TNF-α, and/or IL-2 by effector memory (CD44^+^CD62L^−^), PPD-specific, CD4^+^ T cells, and only BCGΔBCG1419c increased effector memory, PPD-specific CD8^+^ T cell responses in the lungs and spleens compared with unvaccinated mice before challenge. BCGΔBCG1419c increased levels of central memory (CD62L^+^CD44^+^) T CD4^+^ and CD8^+^ cells compared to those of BCG-vaccinated mice. Both BCG strains elicited Th1-biased antigen-specific polyfunctional effector memory CD4^+^/CD8^+^ T cell responses at 10 weeks post-infection, and both vaccines controlled Mtb M2 growth in the lung and spleen. Only BCGΔBCG1419c significantly ameliorated pulmonary inflammation and decreased neutrophil infiltration into the lung compared to BCG-vaccinated and unvaccinated mice. Both BCG strains reduced pulmonary TNF-α, IFN-γ, and IL-10 levels. Taken together, BCGΔBCG1419c increased memory CD8+T cell-associated immunogenicity and mitigated pulmonary inflammation compared with BCG.

## Introduction

Tuberculosis (TB) remains the most widespread and leading cause of mortality for a single bacterial pathogen worldwide. Despite the fact that global TB control efforts, namely, the End TB Strategy, prevent millions of cases and hundreds of thousands of deaths every year, comorbidities related to human immunodeficiency virus infection, diabetes, differences in virulence of clinical isolates, and the emergence of drug-resistant *Mycobacterium tuberculosis* (Mtb) strains have further impeded the elimination of TB, causing a public health problem^1–3^. Bacillus Calmette-Guerin (BCG), the only currently licenced vaccine against TB, confer insufficient protection in adolescents and adults against pulmonary TB^4^. Therefore, numerous efforts have aimed to develop improved TB vaccines in recent decades by employing strategies surveying genes expressed in vivo^5–7^.

To overcome the insufficient protection mediated by BCG, over 20 novel TB vaccine candidates through diverse strategies involving recombinant live mycobacterial vaccines and boosting BCG with subunit vaccines with Mtb antigens have been developed and are at different stages of clinical trials^8^. Two whole-cell-derived vaccine candidates, namely, MTBVAC and VPM1002, have advanced into clinical trials^9–12^. For the development of an improved whole-cell-derived live vaccine against TB, our group developed the BCGΔBCG1419c vaccine candidate by deleting the cyclic di-GMP phosphodiesterase-encoding gene *BCG1419c*. This led to increased in vitro biofilm production by BCGΔBCG1419c compared with its parental strain^13^. We developed this vaccine candidate based on the hypothesis that biofilms of mycobacteria are a feature of the chronic aspect of TB infection^14^. In this regard, last year, biofilm-like structures were reported i*n vivo* in different animal models and human samples^15^, with our perspective on this finding already discussed^16^. Furthermore, it was just shown that aggregated Mtb increased lung pathology in rabbits^17^. We can now hypothesize that BCGΔBCG1419c might protect against these phenotypes (biofilm production and/or infection with aggregated Mtb) and therefore further develop this vaccine candidate.

The originally developed first version of BCGΔBCG1419c harbours a hygromycin resistance marker (Hyg^r^) instead of the *BCG1419c* gene in BCG Pasteur^13^. This vaccine candidate was as safe as its parental BCG in immunocompromised nu/nu mice and improved protection against a high-dose (2.5 × 10^5^ CFU) infection with Mtb H37Rv, accompanied by increased levels of IFN-γ^+^ T lymphocytes in spleens compared with mice receiving BCG^18^, but this version had not evaluated the capacity to elicit multifunctional effector T cells yet in lungs. Moreover, BCGΔBCG1419c controlled bacterial replication upon corticosteroid treatment in a chronic Mtb reactivation model^18^, suggesting that the BCGΔBCG1419c vaccine could be a potential candidate for either preventive or postexposure vaccines. In addition, in a low dose (100 CFUs of Mtb H37Rv) challenge performed with C57BL/6 mice, BCGΔBCG1419c mediated protection during chronic TB infection similarly to parental BCG accompanied by enhanced amelioration of the production of pro-inflammatory cytokines and reduced lung pathology^19^. In line with this information, reduced pneumonia was markedly observed in BCGΔBCG1419c-vaccinated type 2 diabetes (T2D) BALB/c mice compared with that of nonvaccinated mice, displaying superiority to BCG^20^.

Based on these results, we developed a second-generation version of BCGΔBCG1419c, which is devoid of Hyg^r^, now in BCG Pasteur ATCC 35734. The antibiotic-less BCGΔBCG1419c maintains the differential production of cellular antigenic proteins first described for this vaccine candidate^21^, while it also increases the secretion of Tuf, GroEL1, DnaK and GroES compared with that of parental BCG^22^. Moreover, the new BCGΔBCG1419c was safer than parental BCG, as evidenced by the attenuated features both in vitro and in vivo^23^. Furthermore, improved efficacy was achieved by BCGΔBCG1419c vaccination, resulting in a reduction in pulmonary and extrapulmonary TB pathology in a guinea pig model upon infection with a very low dose (10–20 CFUs) of Mtb H37Rv^23^.

For further development of new vaccines against TB, some recommendations have been provided to better ascertain their efficacy in the context of varying factors, such as exposure to environmental mycobacteria, pathogens, and host genetics^24^. Evaluation of the efficacy against different Mtb strains should be considered, as the efficacy of BCG against TB varies geographically, and different Mtb strains elicit differential immune responses with different levels of virulence^8,25,26^.

Since animal models are often challenged with laboratory-adapted strains such as H37Rv or Erdman strains in the preclinical step of vaccine development, it might be challenging to evaluate vaccine efficacy in regions where patients are predominantly infected with different clinical strains of Mtb^27,28^. Moreover, most preclinical studies in mice regarding efficacy of novel TB vaccine candidates focus on determining Mtb replication in lungs only at 4 weeks post-infection (p.i.), with very limited candidates being evaluated for extended protection, except for VPM1002^29^ and BCGΔBCG1419c^18,19^. In this regard, it is worth noting that in C57BL/6 mice, vaccination with BCG Pasteur significantly reduced lung CFU of 9 different Mtb strains at 4 weeks p.i., but that this reduction started to wane at later time points^30^. For these reasons, in the current study, we evaluated the protective efficacy of the second-generation vaccine candidate BCGΔBCG1419c as a vaccine against chronic infection with the Mtb clinical strain M2 from the Haarlem family, for which BCG-derived protection was not significantly achieved^31^, including its parental BCG and unvaccinated controls, at 10 weeks post-infection to determine long-term efficacy of protection. Our results show that BCGΔBCG1419c-mediated protection against Mtb clinical isolate M2 infection is accompanied by superior control of pulmonary inflammation compared to that of unvaccinated- and/or BCG-vaccinated mice.

## Results

### Vaccination with BCGΔBCG1419c maintains antigen specific CD4^+^ T cell responses but increases CD8^+^ T cell responses compared with BCG

**Figure 1:**
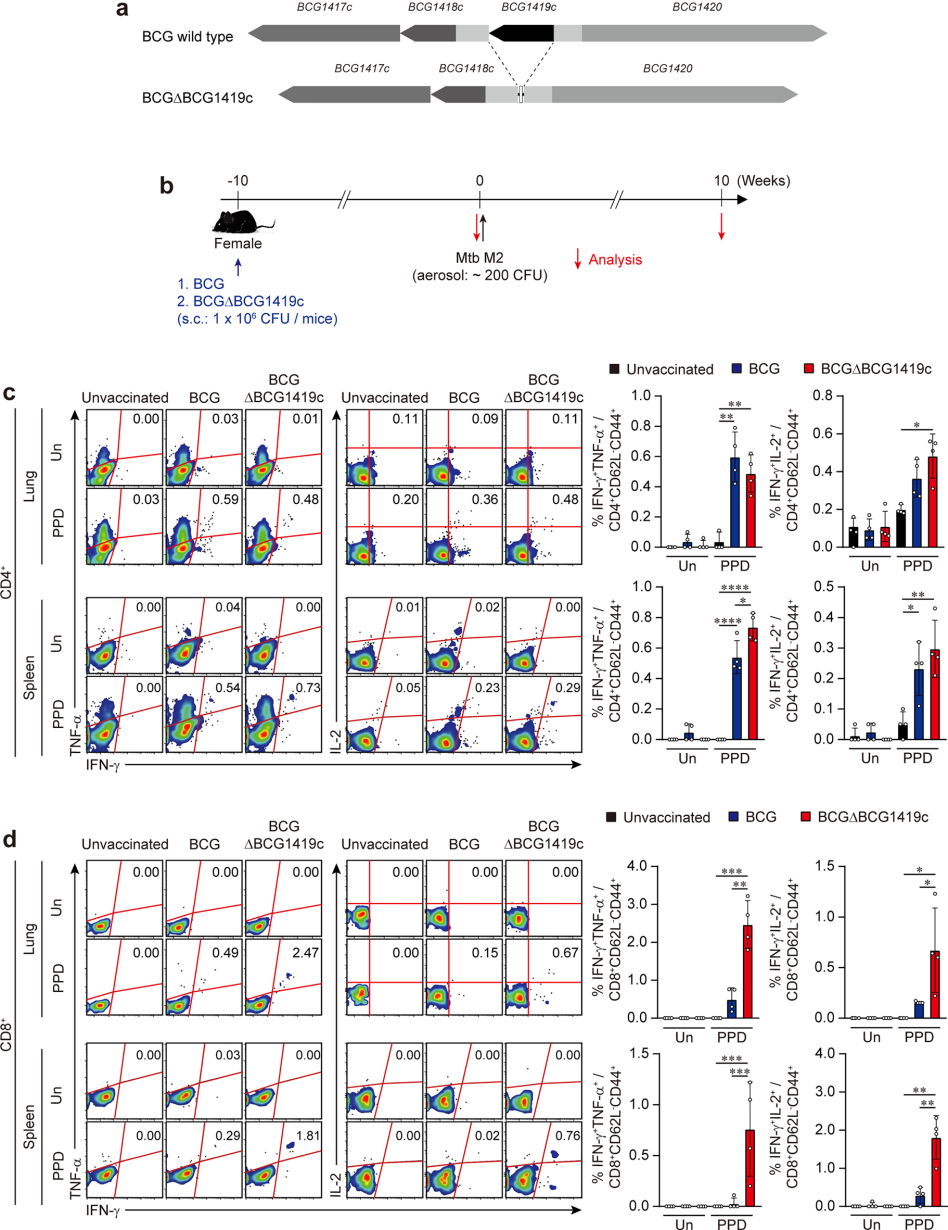
Analysis of antigen specific CD4^+^/CD8^+^ T cell responses induced by vaccination in the lung and spleen. (**a**) Schematic illustration of the gene deletion produced in the second-generation version of BCGΔBCG1419c compared with wild type BCG. (**b**) Experimental scheme for BCGΔBCG1419c and BCG vaccines testing. Each group of female mice (*n* = 10) was vaccinated with BCG or BCGΔBCG1419c via subcutaneous injection (1.0 × 10^6^ CFUs/mouse; blue arrow). Ten weeks after vaccination, mice were aerogenically infected with Mtb strain M2, achieving an initial infectious dose of ~ 200 CFUs (black arrow). Immunological analysis was conducted before and after Mtb infection (red arrow). Bacterial counts and histopathological analysis in each subset of mice were evaluated at the indicated time point after Mtb infection (red arrow). (**c**) Ten weeks after vaccination, mice from each group (*n* = 4) were sacrificed, and cells from lung and spleen were stimulated ex vivo with or without PPD (5 μg/ml) at 37 °C for 9 h in the presence of GolgiPlug. The frequencies of PPD-specific IFN-γ^+^TNF-α^+^- or IFN-γ^+^IL-2^+^-producing CD4^+^CD62L^-^CD44^+^ T-cells were determined by intracellular cytokine staining in the lungs and spleen of each vaccinated mouse and presented as dot plots with bar graphs. (**d**) The frequencies of PPD-specific IFN-γ^+^TNF-α^+^- or IFN-γ^+^IL-2^+^-producing CD8^+^CD62L^-^CD44^+^ T-cells were determined by intracellular cytokine staining in the lungs and spleen of each vaccinated mouse and presented as dot plots with bar graphs. The experimental results are presented as the mean ± SD from 4 mice from each group. The representative mean values are denoted in each FACS plot. One-way ANOVA with post hoc Tukey’s multiple comparison test was used to evaluate the significance. *p < 0.05, **p < 0.01, and ***p < 0.001. The experimental results of one representative experiment are shown.

To evaluate the immunogenicity of the second-generation version of the BCGΔBCG1419c vaccine candidate, mice were vaccinated with either BCG or BCGΔBCG1419c (Fig. 1a, b). To elucidate the mycobacteria-specific CD4^+^/CD8^+^ T cells producing IFN-γ, TNF-α, and IL-2 in both the lung and spleen, isolated single cells from vaccinated mice were stimulated with PPD followed by intracellular cytokine staining. When lymphocytes were stimulated ex vivo with PPD, the frequency of PPD-specific CD4^+^CD62L^-^CD44^+^ T cells with IFN-γ^+^TNF-α^+^ was significantly increased in the lung and spleen from both BCG- and BCGΔBCG1419c-vaccinated mice compared to those of unvaccinated mice (lung: *p* = 0.0012 unvaccinated versus BCG-vaccinated, *p* = 0.0048 unvaccinated versus BCGΔBCG1419c-vaccinated; spleen: *p* < 0.0001 unvaccinated versus BCG-vaccinated, *p* < 0.0001 unvaccinated versus BCGΔBCG1419c-vaccinated). Also, in the spleen, an increased frequency of PPD-specific CD4^+^CD62L^-^CD44^+^IFN-γ^+^TNF-α^+^ T cells in BCGΔBCG1419c-vaccinated mice were observed compared with that of BCG-vaccinated mice (*p* = 0.0186 BCG-vaccinated versus BCGΔBCG1419c-vaccinated).

In addition, BCGΔBCG1419c-vaccinated mice had significantly higher frequency of PPD-specific CD4^+^CD62L^-^CD44^+^ IFN-γ^+^IL-2^+^ T cells compared with unvaccinated mice (lung: *p* = 0.0114 unvaccinated versus BCGΔBCG1419c-vaccinated; spleen: *p* = 0.0222 unvaccinated versus BCG-vaccinated, *p* = 0.0038 unvaccinated versus BCGΔBCG1419c-vaccinated) (Fig. 1c). Notably, unlike the similar induction of PPD-specific CD4^+^ T cells afforded by both BCG strains, the frequency of PPD-specific CD8^+^CD62L^-^CD44^+^ T cells producing IFN-γ, TNF-α and/or IL-2 was markedly increased in both organs compared to the levels in unvaccinated and BCG-vaccinated mice when the mice received BCGΔBCG1419c (IFN-γ^+^TNF-α^+^ in lung: *p* = 0.0009 unvaccinated versus BCGΔBCG1419c-vaccinated, *p* = 0.0011 BCG-vaccinated versus BCGΔBCG1419c-vaccinated; IFN-γ^+^IL-2^+^ in lung: *p* = 0.0212 unvaccinated versus BCGΔBCG1419c-vaccinated, *p* = 0.0495 BCG-vaccinated versus BCGΔBC1419c-vaccinated; IFN-γ^+^TNF-α^+^ in spleen: *p* = 0.0001 unvaccinated versus BCGΔBCG1419c-vaccinated, *p* = 0.0005 BCG-vaccinated versus BCGΔBC1419c-vaccinated; IFN-γ^+^IL-2^+^ in spleen: *p* = 0.008 unvaccinated versus BCGΔBCG1419c-vaccinated, *p* = 0.01 BCG-vaccinated versus BCGΔBC1419c-vaccinated) (Fig. 1d). In addition, BCGΔBCG1419c-vaccinated mice displayed higher frequencies of central memory T cell phenotype (CD4^+^/CD8^+^CD62L^+^CD44^+^) in the lung after vaccination compared to those of BCG-vaccinated mice (CD4^+^CD62L^+^CD44^+^ in lung: *p* = 0.001 BCG-vaccinated versus BCGΔBCG1419c-vaccinated; CD8^+^CD62L^+^CD44^+^ in lung: *p* = 0.0127 BCG-vaccinated versus BCGΔBCG1419c-vaccinated) (Supplementary Fig. S1). These findings indicated that BCGΔBCG1419c-vaccinated mice induced comparable antigen-specific CD4^+^ T cell responses accompanied by enhanced antigen-specific CD8^+^ T cell responses as well as increased levels of central memory T CD4^+^ and CD8^+^ cells compared to those of BCG-vaccinated mice.

### Vaccination of mice with BCGΔBCG1419c reduced the bacterial loads after challenge with Mtb strain M2, similar to BCG

**Figure 2:**
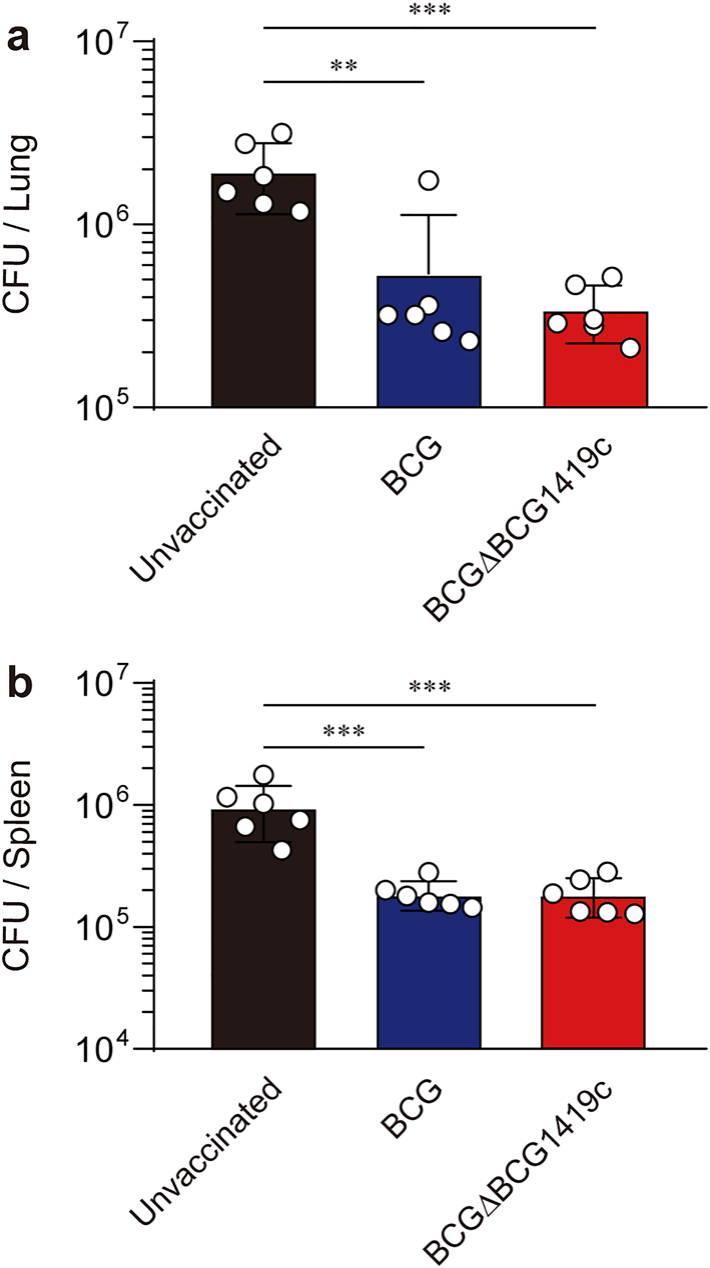
Long-term protective efficacy against replication of Mtb strain M2 with BCG and BCGΔBCG1419c vaccination. (**a**, **b**) CFUs in the lungs and spleens of each subset of mice (*n* = 6/group) at 10 weeks post-infection were assessed by enumerating viable bacteria. Fold change was presented by comparing the mean CFU values between group. One-way ANOVA with post hoc Tukey’s multiple comparison test was used to evaluate the significance. **p < 0.01 and ***p < 0. 001. The experimental results of one representative experiment are presented.

Ten weeks postvaccination with BCG and BCGΔBCG1419c, mice were aerogenically infected with 200 CFUs of the Mtb clinical strain M2^31^. Then, the mice were euthanised at 10 weeks post-infection to quantitate the bacterial loads in the lungs and spleens (Fig. 1b). Vaccination with BCGΔBCG1419c reduced the mean bacterial loads in the lungs to a greater extent than that of BCG-vaccinated mice compared to unvaccinated mice (*p* = 0.0022 unvaccinated versus BCG-vaccinated, 3.63-fold reduction; *p* = 0.0007 unvaccinated versus BCGΔBCG1419c-vaccinated, 5.68-fold reduction), although there was no significant difference between the mice vaccinated with either BCG strain (Fig. 2a). In line with this result, a similar reduction in bacterial loads in the spleens from both groups of vaccinated mice was observed without displaying protective superiority between the vaccinated groups (*p* = 0.0005 unvaccinated versus BCG-vaccinated; *p* = 0.0005 unvaccinated versus BCGΔBCG1419c-vaccinated) (Fig. 2b). Collectively, both vaccines were effective in conferring protection with reduced bacterial loads in the lungs and spleens against Mtb strain M2 infection at 10 weeks post-infection.

### Durable antigen specific CD4^+^/CD8^+^ polyfunctional T cells in the lung were induced by both vaccinations at 10 weeks post-infection

**Figure 3:**
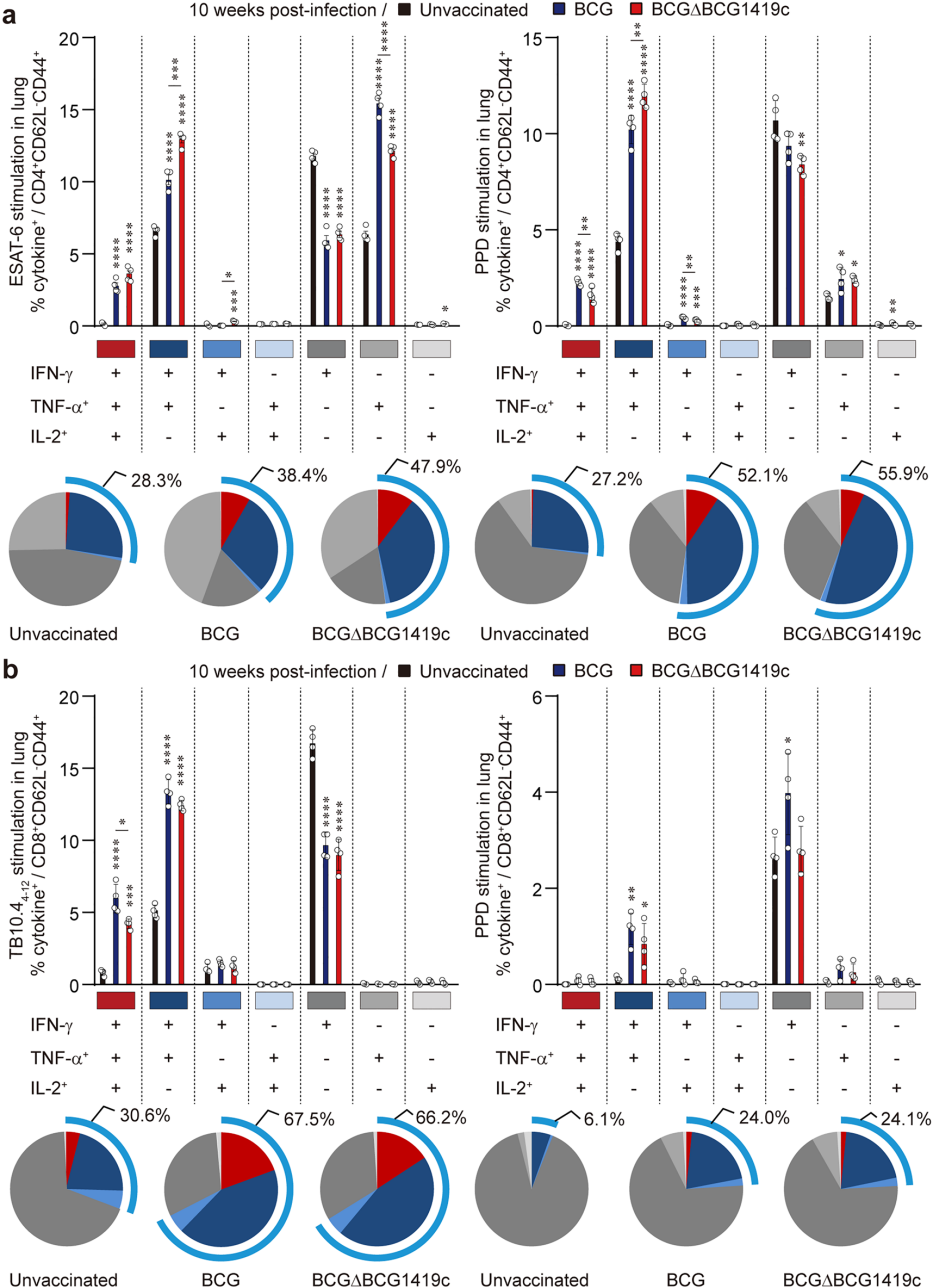
Qualitative analysis of antigen-specific polyfunctional CD4^+^/CD8^+^ T cell responses in the lungs of BCG- and BCGΔBCG1419c-vaccinated mice following infection with Mtb strain M2. Mice (*n* = 6/group) were sacrificed at 10 weeks post-infection, and the lung cells were stimulated ex vivo with the indicated antigens at 37 °C for 9 h in the presence of GolgiPlug. (**a**) ESAT-6 (1 μg/ml)- or PPD (5 μg/ml)-stimulated lung cells from each vaccinated group were assessed based on the percentage of total CD4^+^CD62L^-^CD44^+^ T cells with different combinations of cytokine production and are presented as dot plots with bar graphs (**a**, upper). The pie charts represent the fractions of CD4^+^CD62L^-^CD44^+^ T cell producers of IFN-γ, TNF-α and IL-2 in each vaccinated group (3**a**, lower). The blue arc denotes the percentage of cytokine-positive T cells (IFN-γ^+^TNF-α^+^IL-2^+^-, IFN-γ^+^TNF-α^+^-, IFN-γ^+^IL-2^+^-, and TNF-α^+^IL-2^+^-CD4^+^CD62L^-^CD44^+^ T cells). (**b**) TB10.4 (1 μg/ml)- or PPD (5 μg/ml)-stimulated lung cells from each vaccinated group were assessed based on the percentage of total CD8^+^CD62L^-^CD44^+^ T cells with different combinations of cytokine production and are presented as dot plots with bar graphs (**b**, upper). The pie charts represent the fractions of CD8^+^CD62L^−^CD44^+^ T cell producers of IFN-γ, TNF-α and IL-2 in each vaccinated group (**b**, lower). The blue arc denotes the percentage of cytokine-positive T cells (IFN-γ^+^TNF-α^+^IL-2^+^-, IFN-γ^+^TNF-α^+^-, IFN-γ^+^IL-2^+^-, and TNF-α^+^IL-2^+^-CD8^+^CD62L^-^CD44^+^ T cells). Data from one representative experiment are presented as the mean ± SD from pooled samples (*n* = 4) from each group (*n* = 6). One-way ANOVA with post hoc Tukey’s multiple comparison test was used to evaluate the significance. *p < 0.05, **p < 0.01, ***p < 0.001, and ****p < 0.0001.

Accumulating data have suggested that CD4^+^/CD8^+^ T cells producing multiple cytokines, including IFN-γ, TNF-α, and IL-2, are highly associated with protective correlates against TB in various studies, including mouse^31–33^ and human studies^34,35^. To determine whether antigen-specific polyfunctional T cell responses persisted in the vaccinated groups, we next investigated the immune responses in the lungs of a subset of mice after Mtb strain M2 challenge according to the FACS gating strategy (Supplementary Fig. S2). Ex vivo responses to either ESAT-6 or PPD, showed that the frequency of antigen-specific CD4^+^ T cells producing IFN-γ, TNF-α, and/or IL-2 was significantly increased in the lungs of both BCG- and BCGΔBCG1419c-vaccinated mice compared to unvaccinated mice. ESAT-6-specific double-positive CD4^+^ T cells (IFN-γ^+^TNF-α^+^ and IFN-γ^+^IL-2^+^) were significantly increased in BCGΔBCG1419c-vaccinated mice compared to those in BCG-vaccinated mice (IFN-γ^+^TNF-α^+^; *p* = 0.0002 BCG-vaccinated versus BCGΔBCG1419c-vaccinated, IFN-γ^+^IL-2^+^; *p* = 0.018 BCG-vaccinated versus BCGΔBCG1419c-vaccinated) (Fig. 3a). With PPD stimulation, BCG vaccination elicited higher frequencies of CD4^+^ T cells, including IFN-γ^+^TNF-α^+^IL-2^+^ and IFN-γ^+^IL-2^+^, except for IFN-γ^+^TNF-α^+^ cells, than the frequencies with BCGΔBCG1419c vaccination (IFN-γ^+^TNF-α^+^IL-2^+^; *p* = 0.0072 BCG-vaccinated versus BCGΔBCG1419c-vaccinated, IFN-γ^+^IL-2^+^; *p* = 0.009 BCG-vaccinated versus BCGΔBCG1419c-vaccinated, IFN-γ^+^TNF-α^+^; *p* = 0.0069 BCG-vaccinated versus BCGΔBCG1419c-vaccinated) (Fig. 3a).

In addition, similar frequencies of polyfunctional CD8^+^ T cells specific to TB10.4_4–12_ MHC-I-restricted epitope which dominantly elicited IFN-γ-producing CD8^+^ T cells^36^ were detected in both vaccinated group only except for TB10.4_4–12_-specific IFN-γ^+^TNF-α^+^IL-2^+^ response which was significantly induced by BCG compared with BCGΔBCG1419c (IFN-γ^+^TNF-α^+^IL-2^+^; *p* = 0.0113 BCG-vaccinated versus BCGΔBCG1419c-vaccinated) at 10 weeks post-infection (Fig. 3b). These results demonstrated that both BCG strains were effective in inducing PPD- or TB10.4_4–12_-specific polyfunctional T cell responses during chronic infection, although the responses were of a different profile.

### BCGΔBCG1419c is more effective than BCG in ameliorating pulmonary TB pathology accompanied by reduced infiltration of neutrophils

**Figure 4:**
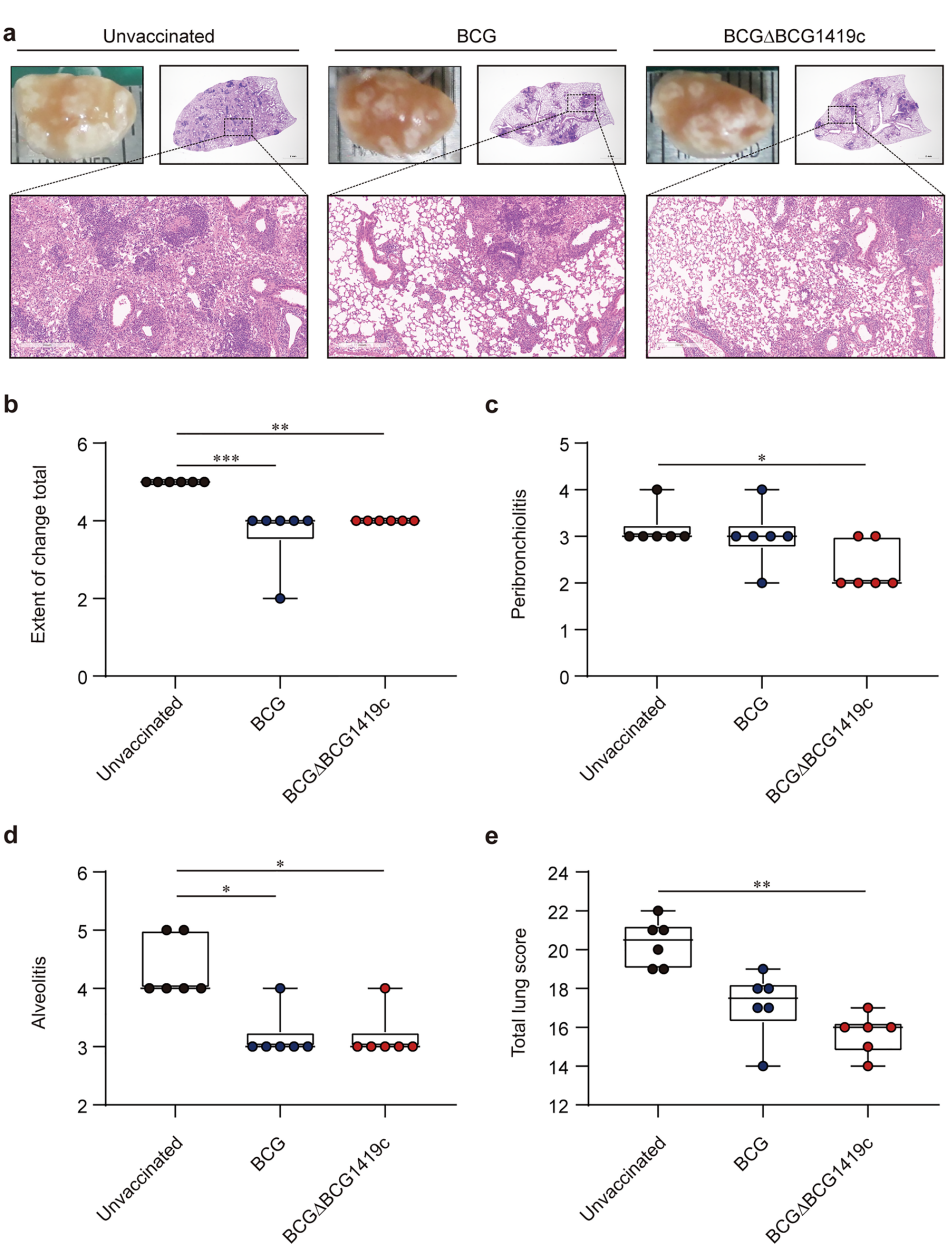
Histopathological assessment of pulmonary inflammation in BCG- and BCGΔBCG1419c-vaccinated mice upon Mtb strain M2 infection. (**a**) The superior lobes of the right lung of each subset of mice (*n* = 6/group) were analysed by H&E staining, and representative lung lobes are displayed as gross images at 10 weeks post-infection (10×: scale bar = 2.0 mm, 100 × : scale bar = 300 μm). (**b–e**) Then, H&E-stained sections were scored for the extent of total change, peribronchiolitis, alveolitis, and total lung score were assessed with the scoring system described in Methods. Data (*n* = 6) from one representative experiment are presented as a box and whisker plot showing all points. Kruskal–Wallis followed by Dunn’s multiple comparison test was used to evaluate the significance. *p < 0.05, **p < 0.01, and ***p < 0.001.

After observing that both BCG and BCGΔBCG1419c conferred protection by controlling Mtb strain M2 replication as well as by inducing somewhat different profiles of polyfunctional T cell responses, we next investigated whether BCGΔBCG1419c possessed the capacity to reduce the levels of structural changes related to lung inflammation in our murine model as it did after low-dose (100 CFUs of Mtb H37Rv) infection of C57BL/6 mice^19^ and very-low-dose (10–20 CFUs of Mtb H37Rv) infection of guinea pigs^23^. Thus, mice from the unvaccinated and vaccinated groups were euthanised at 10 weeks post-infection with Mtb strain M2, and histopathological analysis of the lungs was performed (Fig. 4a). Notably, mice vaccinated with BCGΔBCG1419c exhibited significantly reduced lung tissue damage compared to unvaccinated mice, as evidenced by the peribronchiolitis (*p* = 0.0489), alveolitis (*p* = 0.0107), and total lung scores (*p* = 0.0013). Furthermore, BCG-vaccinated mice did not have a significantly ameliorated total lung score compared to that of unvaccinated mice except for alveolitis (*p* = 0.0107) (Fig. 4b–e). No significant changes were observed between unvaccinated and vaccinated mice with respect to perivasculitis, granuloma formation, and necrosis, factors that also account for the total lung score (Supplementary Fig. S3).

**Figure 5:**
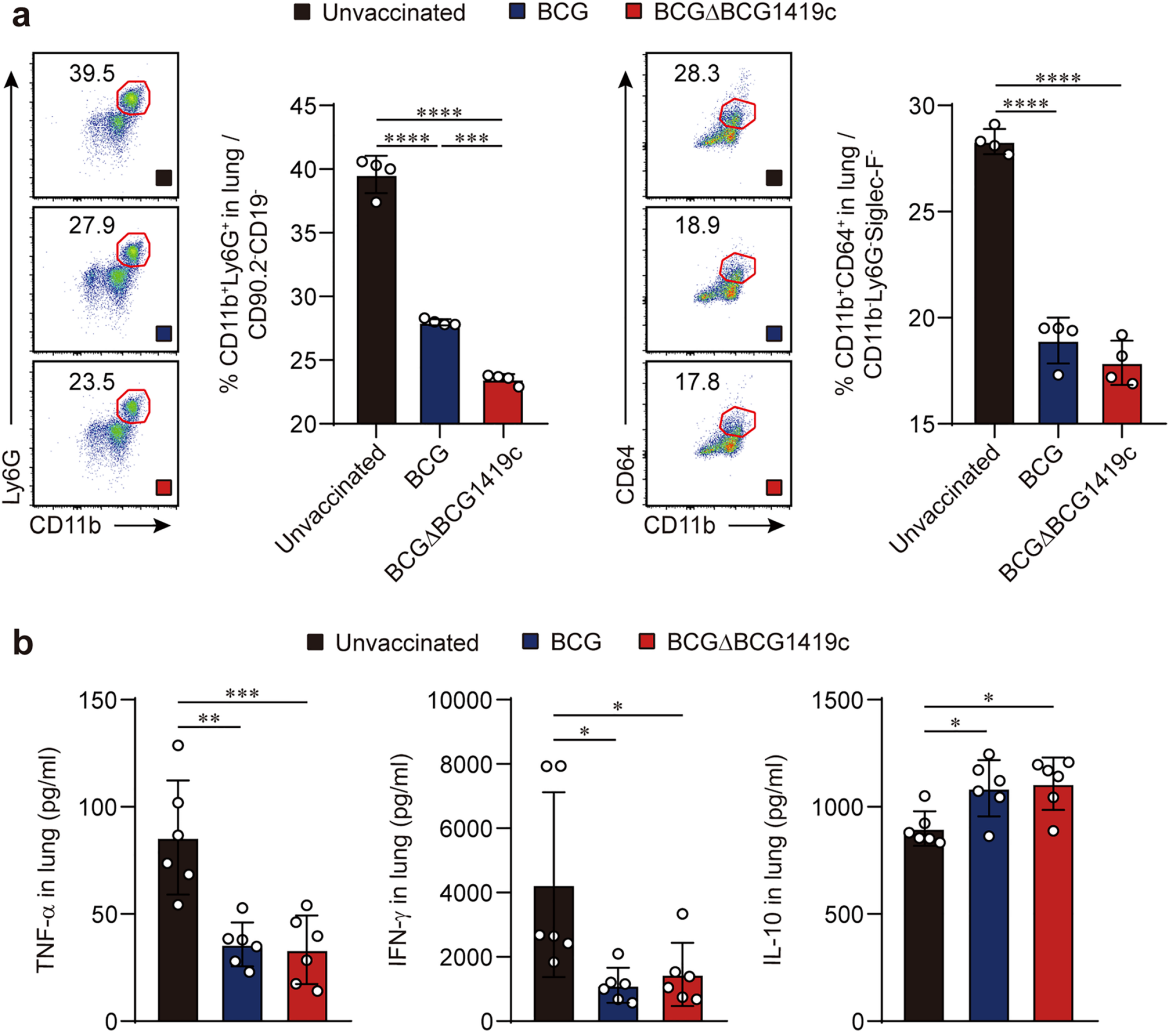
Assessment of local immune responses represented by cytokine production and cellular infiltration in BCG- and BCGΔBCG1419c-vaccinated mice after infection. (**a**) The frequencies of neutrophils and macrophages in the lungs of each group were evaluated at 10 weeks post-infection. The experimental results of one representative experiment are presented as the mean ± SD from pooled samples (*n* = 4) from each group (*n* = 6). One-way ANOVA with post hoc Tukey’s multiple comparison test was used to evaluate the significance. ***p < 0.001, and ****p < 0.0001. (**b**) At 10 weeks post-infection, lung lysates from each group (*n* = 6) were used to quantify the levels of TNF-α, IFN-γ, and IL-10. Data are expressed as the mean ± SD from each group (*n* = 6). One-way ANOVA with post hoc Tukey’s multiple comparison test was used to evaluate the significance. *p < 0.05, **p < 0.01, and ***p < 0.001.

Moreover, cellular infiltration of neutrophils and macrophages into the lungs was investigated by flow cytometry analysis (Supplementary Fig. S4). The frequencies of neutrophils and macrophages in the lung were significantly decreased by vaccination with either BCG strain (neutrophils: *p* < 0.0001 unvaccinated versus BCG, *p* < 0.0001 unvaccinated versus BCGΔBCG1419c; macrophages: *p* < 0.0001 unvaccinated versus BCG, *p* < 0.0001 unvaccinated versus BCGΔBCG1419c), whereas vaccination with BCGΔBCG1419c reduced neutrophils more than BCG did (*p* = 0.0002 BCG versus BCGΔBCG1419c) (Fig. 5a). To further explore the effect of vaccination on pulmonary inflammation, we assessed the secretion of TNF-α, IFN-γ, and IL-10 in the lungs of unvaccinated and vaccinated mice. Both BCG and BCGΔBCG1419c vaccinations significantly reduced the pro-inflammatory cytokines TNF-α (*p* = 0.001 unvaccinated versus BCG; *p* = 0.0007 unvaccinated versus BCGΔBCG1419c) and IFN-γ (*p* = 0.0214 unvaccinated versus BCG; *p* = 0.0404 unvaccinated versus BCGΔBCG1419c), whereas IL-10 was increased by both vaccinations compared to the levels in unvaccinated mice (*p* = 0.0299 unvaccinated versus BCG; *p* = 0.0159 unvaccinated versus BCGΔBCG1419c) (Fig. 5b). Taken together, these results demonstrate that vaccination of mice with BCGΔBCG1419c conferred improved amelioration of pulmonary inflammation against Mtb strain M2 compared with that of vaccination with BCG.

## Discussion

Given the reduced protection against pulmonary TB in adult humans provided currently by current BCG, among other candidates, two live mycobacteria, namely, VPM1002 and MTBVAC, have advanced into clinical trials. However, it should be noted that variations in their protective efficacies relative to animal strains, infection duration, and Mtb challenge strain in the preclinical setting were found. For example, when infected with 150–200 CFUs of Mtb H37Rv, VPM1002 showed protection equal to that of BCG Danish in reducing Mtb loads in the lungs at 30 days post-infection, while it was more protective than BCG after 60 days post-infection in C57BL/6 mice, an effect associated with central memory CD4^+^ T cells^37^. VPM1002 significantly reduced Mtb loads in BALB/c mice up to 90 days post-infection compared with those of BCG-vaccinated mice, and it protected mice infected with 200 CFUs of Mtb W/Beijing up to 200 days post-infection, whereas BCG did not protect at all^29^. How well VPM1002 would protect C57BL/6 mice against other Mtb strains, or what its efficacy would be compared with BCG Pasteur, to mention an alternative BCG strain, have not been reported. Further to this, whether VPM1002 also induces an increased central memory CD8^+^ T cell response, as observed here for BCG∆BCG1419c (Supplementary Fig. S1) remains to be determined. We elaborate more on some remaining questions in the next few paragraphs.

MTBVAC conferred similar protection to that afforded by BCG Danish in C57BL/6 and BALB/c mice, while it improved protection over BCG only in C3H/HeNRj mice, when mice were intranasally infected with 20 CFUs of Mtb H37Rv and lung loads were determined at 4 weeks post-infection^38^. In a separate study, MTBVAC protected C3H/HeNRj mice as equally well as BCG Pasteur when challenged with Mtb H37Rv, while it outperformed this BCG strain when Mtb Beijing W4 was used for infection, based on the reduction of Mtb loads in the lung at 4 weeks post-infection^39^. How MTBVAC protects mice at time points longer than 4 weeks has not been reported. Additionally, why does the efficacy of MTBVAC vary when compared to those of BCG Danish or Pasteur? Would the efficacy of VPM1002 also vary if compared to that of BCG Pasteur? Partly because of these questions, as well as recent reports showing biofilm-like structures in vivo^15^ and increased virulence of aggregated Mtb in rabbits^17^, we have continued developing BCGΔBCG1419c, as we think that there could be populations that would be best protected with a different and novel TB vaccine candidate and that one potential replacement would not necessarily have universal application. Furthermore, the evaluation of a potential improved protection against biofilm-like structures produced in vivo afforded by BCGBCG1419c compared with BCG seems now more technically feasible.

In the present study, we investigated the protective efficacy of a second-generation version of the BCGΔBCG1419c vaccine candidate against infection with the Mtb clinical M2 strain as a preventative vaccine regimen. BCGΔBCG1419c elicited T cell memory responses represented by the robust induction of antigen-specific polyfunctional effector memory CD4^+^/CD8^+^ CD44^+^CD62L^-^ T cell responses before (Fig. 1) and after infection (Fig. 3), and it especially induced CD8^+^ T cells more than BCG did prior to challenge. In addition, BCG∆BCG1419c-immunized mice displayed enhanced induction of both central memory T CD4^+^ and CD8^+^ cells in the lung after immunization (Supplementary Fig. S1), whereas VPM1002 increased only central memory T CD4^+^ responses^37^. Effector memory and central memory T cells are thought to play different roles in protection against TB^40^. Here we observed that BCG∆BCG1419c was particularly effective in triggering effector memory polyfunctional T cell responses compared with BCG, before and after infection. Considering, for instance, that chronic protection against TB conferred by VPM1002 was associated with central memory CD4^+^ T cells^37,41^, it was initially anticipated that BCG∆BCG1419c could mediate improved protection compared to parental BCG against Mtb infection. However, similar bacterial reduction was observed at 10 weeks post-infection. This may be partly because BCG∆BCG1419c immunization-derived antigenic repertoire in memory T cells may not mediate improved protection against Mtb M2 strain compared to parental BCG, implying that vaccine candidates should be tested against various Mtb strains harbouring differential antigenic profiles for universal future application. An alternative explanation is that the increased level of central memory T cells observed for BCG∆BCG1419c-vaccinated mice compared to those receiving BCG would impact efficacy under settings different to those tested here (e.g., vaccine or strain dose/route, animal model employed, etc.).

The BCGΔBCG1419c vaccine candidate is devoid of the c-di-GMP phosphodiesterase gene *BCG1419c*, which is required for the degradation of c-di-GMP. Of note, this second messenger is associated with biofilm formation, virulence, and differentiation of bacteria^41,42^. C-di-GMP of bacterial origin has been reported to be the ligand for stimulator of interferon (IFN) genes (STING) that signals via the tank-binding kinase-1 (TBK1)-interferon regulatory factor 3 (IRF3) cascade to produce type I IFN- and NF-κB-mediated cytokines^43,44^. Furthermore, these STING agonists have shown potential use as novel vaccine adjuvants, as evidenced by their immunostimulatory properties, due to their ability to increase antigen-specific T cell and humoral responses^45,46^. In addition, Lu et al.^47^ demonstrated that CD8^+^ T cells are required for the optimal protective immune response to inhibit Mtb growth by coordinating with CD4^+^ T cells. Although experimental evidence has not been provided, we hypothesise that BCGΔBCG1419c might increase its production of c-di-GMP, resulting in the improved induction of CD8^+^ T cells pre-infection^48^; however, future investigation is required to determine whether BCG∆BCG1419c immunization-derived superior effector memory CD8^+^ T cell responses may play a marginal role or not considering that similar bacterial reduction was observed in BCG- and BCG∆BCG1419c-immunized mice.

Regarding the efficacy of protection, BCGΔBCG1419c was effective in controlling the replication of the M2 clinical Mtb strain in the lung to the same extent as the parental BCG and more effectively than unvaccinated mice (Fig. 2). Moreover, a significant reduction in pulmonary inflammation (Fig. 4) accompanied by decreased infiltration of neutrophils to the lungs provided only by BCGΔBCG1419c was observed compared to those of BCG-vaccinated and unvaccinated mice (Fig. 5). Our results further support the fact that the new version of BCGΔBCG1419c significantly reduces pulmonary inflammation accompanied by decreased infiltration of neutrophils to the lung, to levels greater than those attained by vaccination with BCG in C57BL/6 mice, despite the fact that (1) twofold the dose of Mtb strain tested previously was used^19^ and (2) an Mtb clinical isolate was employed for which BCG Pasteur was shown to be ineffective^31^.

These variable efficacies of protection may partly be attributed to the insufficient understanding of mycobacterial strain diversity and their impact in infection outcome^49^. Homolka et al. and our group have suggested that diverse Mtb strains are required for vaccine testing considering the genetic diversity in Mtb strains^24,50^. In the Haarlem lineage of Mtb, including Mtb M2, the absence of *Rv1354c* has been reported^51^. *Rv1354c* and *Rv1357c* are involved in c-di-GMP metabolism, and BCG harbours the homologues *BCG1416c* and *BCG1419c* to these genes, respectively. Given that BCGΔBCG1416c displayed some differences in mediating immune responses and protection against Mtb infection compared to those of BCGΔBCG1419c^21^, it is conceivable that Mtb M2 lacking *Rv1354c* infection may be able to affect vaccine efficacy provided by BCGΔBCG1419c, although the underlying mechanisms should be elucidated in a future study.

During Mtb infection, the induction of multifunctional antigen-specific T cells by vaccination is important for protection against Mtb infection^35,52^. Along with the similar protection mediated by both BCG and BCGΔBCG1419c, these two vaccines elicited sustained antigen-specific polyfunctional effector memory CD4^+^/CD8^+^ CD44^+^ CD62L^-^ T cells coproducing IFN-γ, TNF-α and/or IL-2 during chronic TB infection. Conversely, other researchers have reported an imperfect correlation between polyfunctional T cells and protective efficacy. For instance, BCG boosted with an Ad5 vector expressing Ag85A via the intradermal route induced polyfunctional CD4^+^ T cells in the spleen of mice, resulting in no correlation with vaccine-derived protection^53^. In another study, VPM1002 boosted with MVA85A mediated better protection compared to BCG boosted with MVA85A, which elicited increased levels of CD4^+^ T cell polyfunctionality^54^. Moreover, a lack of correlation between the development of TB and the magnitude of CD4^+^ T cell polyfunctionality has been reported in humans^55,56^. In our data, mice vaccinated with either BCG or BCG∆BCG1419c displayed an increased frequency of TNF-α^+^ single-positive CD4^+^ T cells upon both ESAT-6 and PPD restimulation compared to the frequencies in unvaccinated mice (Fig. 3), indicating that the high induction of antigen-specific multifunctional T cells itself might not fully address vaccine-derived protection. Therefore, additional mechanisms contributing to protective immunity should be investigated above and beyond T cell functionality.

We observed that both BCG and BCGΔBCG1419c reduced the levels of the pro-inflammatory cytokines TNF-α and IFN-γ with increased production of the anti-inflammatory cytokine IL-10 in the lung compared to those in unvaccinated mice. Notably, mice vaccinated with BCGΔBCG1419c exhibited significantly decreased infiltration of neutrophils in the lung compared to that of unvaccinated and BCG-vaccinated mice. Collectively, considering that immunopathology is highly associated with granulocytic influx^57,58^, BCGΔBCG1419c may play an important role in regulating granulocyte-mediated pulmonary pathology, and further studies may be required whether BCG∆BCG1419c might directly affect granulocyte influx via chemokine regulation. Interestingly, when C57BL/6 mice vaccinated with BCGΔBCG1419c were challenged with Mtb H37Rv, IL-10 was reduced^19^, as opposed to the induction observed here upon infection with the Mtb M2 strain, therefore strengthening the notion that different Mtb strains may require a different vaccine for improved protection.

As we have already shown that during chronic TB infection produced by H37Rv in C57BL/6 mice^19^, BCGΔBCG1419c significantly reduced pulmonary IL-6 and TNF-α, it could be that this effect contributes to the reduced inflammation reported here. This may be associated, at least to some extent, to the differential production of antigenic proteins by BCGΔBCG1419c compared with BCG^21,22^, which could elicit immune responses where mediators other than the ones already discussed above, could be involved.

Our current study has certain limitations that deserve further consideration. First, unlike our previous report, we found that wild-type BCG conferred protection against Mtb M2 infection^31^. Potential explanations include the following: (1) the inoculation dose of BCG was different between the work of Gröschel et al.^59^ and the current study, as it has been reported that the efficacy and immunogenicity were differentially affected according to the BCG dose. (2) This could also be the result of using different parental BCG strains (ATCC 35734 in this study versus BCG Pasteur 1173P2 kindly provided by Dr Brosch). Of note, we observed a similar reduction in Mtb M2 in infected organs in mice vaccinated with BCGΔBCG1419c or BCG (approx. 0.8−log_10_ reduction).

For enhanced protection provided by BCGΔBCG1419c to become more evident, it could be that an infection time longer than 10 weeks needs to be evaluated. However, we acknowledge that in C57BL/6 mice infected with Mtb H37Rv, protection against replication at 6 months post-infection was similar to that afforded by BCG (approx. 1−log_10_ reduction), where pulmonary inflammation was reduced only upon vaccination with BCGΔBCG1419c^19^. This would potentially rule out the need to wait for a longer time to achieve an increased effect on the reduction of Mtb replication after vaccination with BCGΔBCG1419c. Therefore, we think these findings point towards a possible “saturation” effect, whereby adult C57BL/6 mice subcutaneously vaccinated with BCG or any whole, live attenuated vaccine candidate cannot further reduce the Mtb load when infected with 10^2^ CFUs of any Mtb strain below 1−log_10_. In support of this notion, VPM1002 reduced Mtb H37Rv loads in the lungs of C57BL/6 mice by 0.8-log_10_ compared with that of nonvaccinated controls^37^, while MTBVAC reduced the lung loads of Mtb H37Rv by approximately 1−log_10_ at 4 weeks post-infection^38^. We acknowledge that other reports have shown a greater than 1−log_10_ CFU reduction in Mtb burden in C57BL/6 mice^60,61^. However, for instance, the work by Heijmenberg, et al.^60^ had several differences compared to ours: (a) they found an increased efficacy against a Mtb Beijing strain, but not against H37Rv, (b) said increased effect in reducing Mtb Beijing loads was observed only after intratracheal vaccination, not with subcutaneous vaccination (as we used here), and (3) the infectious dose they employed was close to 20–50 CFU (four- to ten-fold less than our work). Regarding the work by Khan et al.^61^ their increased drop in Mtb burden was found when adjuvants were used, and this was observed at 30 days (not 10 weeks) post-infection. Overall, our data show that the second-generation BCGΔBCG1419c confers protection against the Mtb clinical isolate M2 by ameliorating lung inflammation with decreased infiltration of neutrophils during chronic TB. These findings coupled with our previous reports, provide the rationale for the continued investigation of BCGΔBCG1419c for its optimal application.

## Methods

### Ethical statement

All animal studies were carried out according to the guidelines of the Korean Food and Drug Administration (KFDA). The experimental protocols used in this study were reviewed and approved by the Ethics Committee and Institutional Animal Care and Use Committee (Permit Number: 2020-0126) of the Laboratory Animal Research Center at Yonsei University College of Medicine (Seoul, Korea). All experiments complied with the ARRIVE guidelines.

### Mice

Specific pathogen-free female C57BL/6J mice (6–7 weeks old) were purchased from Japan SLC, Inc. (Shizuoka, Japan) and maintained under barrier conditions in the ABSL-3 facility at the Yonsei University College of Medicine. The animals were fed a sterile commercial mouse diet with ad libitum access to water under standardised light-controlled conditions (12-h light and 12-h dark periods). The mice were monitored daily, and none of the mice showed any clinical signs or illness during this experiment.

### Preparation of *Mycobacterium* spp

Mycobacterial strains included the *M. bovis* BCG Pasteur ATCC 35734 (hereafter referred to as BCG), its isogenic derivative, second-generation *M. bovis* BCGΔBCG1419c^22,23^, and Mtb strain M2 from the International Tuberculosis Research Center (ITRC, Changwon, Gyeongsangnam-do, Korea)^31^. These strains were cultured in Middlebrook 7H9 broth (Difco Laboratories, Detroit, MI, USA) supplemented with 0.02% glycerol and 10% (vol/vol) oleic acid-albumin-dextrose-catalase (OADC, Becton Dickinson, Sparks, MD, USA) for 28 days at 37 °C. Single-cell suspensions of each strain were prepared as previously described^62^.

### Vaccination and challenge protocol

Mice were vaccinated with BCG or BCGΔBCG1419c via subcutaneous injection (1.0 × 10^6^ CFUs/mouse). Ten weeks after vaccination, the vaccinated mice were aerogenically challenged with the Mtb M2 strain as previously described^31^. Aerosol infection was performed using a Glas-Col aerosol apparatus (Terre Haute, IN, USA) adjusted to achieve an initial infectious dose of 200 CFUs. At 10 weeks postchallenge, mice from each group were euthanised for analysis of the bacterial load, histopathology, and immunological assays, including the frequency of multifunctional T cells and infiltrating myeloid cells.

### Bacterial enumeration

At 10 weeks following Mtb challenge, six mice per group were euthanised with CO_2_, and lungs and spleens were homogenised. The number of viable bacteria was determined by plating serial dilutions of the organ homogenates onto Middlebrook 7H11 agar (Difco, USA) supplemented with 10% OADC (Difco, USA) and amphotericin B (Sigma-Aldrich, USA). Colonies were enumerated after 4 weeks of incubation at 37 °C.

### Flow cytometry and intracellular cytokine staining

For T cell analysis, single-cell suspensions (1.0 × 10^6^ cells) of the lungs and spleens of unvaccinated or vaccinated mice were stimulated with PPD (Purified Protein Derivative) (5 μg/ml) or ESAT-6 (1 μg/ml) at 37 °C for 9 h in the presence of GolgiPlug (BD Biosciences). TB10.4_4-12_ (IMYNYPAML; 1 μg/ml, synthesised from Peptron, Daejeon, South Korea) was used for analysing CD8^+^ T cells. The recombinant ESAT-6 protein was produced as previously described^63^. PPD was kindly provided by Dr Michael Brennan at Aeras (Rockville, MD, USA). PPD was used for assessing BCG-induced immune responses, TB10.4_4–12_ was employed to further compare the functionality of CD8^+^ T cells between BCG wild type and BCGΔBCG1419c, and ESAT-6 was used for ex vivo stimulation to test whether Mtb (ESAT-6)-specific T cell responses can be modulated or affected by each BCG vaccination. Cells were first washed with 2% FBS containing PBS and blocked with anti-CD16/32 (BioLegend, RRID: AB_1574975) at 4 °C for 20 min. After the cells were stained with LIVE/DEAD™ Fixable Viability Dye eFluor™ 780 (Thermo Fisher Scientific), the surface was stained with peridinin chlorophyll (PerCP)-Cy5.5-conjugated anti-CD4 (RRID:AB_393977), Brilliant Violet (BV) 786-conjugated anti-CD8a (RRID:AB_2721167), BV421-conjugated anti-CD44 (RRID:AB_1645273), and Alexa Fluor 700-conjugated anti-CD62L (RRID:AB_1645210) (BD Biosciences) antibodies at 4 °C for 30 min and washed. These cells were permeabilised and fixed with the Cytofix/Cytoperm kit (BD Biosciences) at 4 °C for 30 min. Then, the cells were washed twice with Perm/Wash (BD Biosciences) and intracellularly stained with phycoerythrin (PE)-conjugated anti-IFN-γ (RRID:ΑΒ_315402), allophycocyanin (APC)-conjugated anti-TNF-α (RRID:AB_315429), and PE-Cy7-conjugated anti-IL-2 (RRID:AB_2561750) (BioLegend, San Diego, CA, USA) at 4 °C for 30 min. After washing three times with Perm/Wash, the cells were fixed with IC Fixation buffer (eBioscience). To dissect the lung-infiltrated myeloid cells, namely, neutrophils and macrophages, single-cell suspensions (1.0 × 10^6^ cells) of the lungs from unvaccinated or vaccinated mice were first washed with 2% FBS containing PBS and blocked with anti-CD16/32 (BioLegend, RRID:AB_1574975) at 4 °C for 20 min. The cells were stained with LIVE/DEAD™ Fixable Far Red Dead Cell Stain Kit (Thermo Fisher Scientific) and then surface stained with BV605-conjugated anti-CD90.2 (RRID:AB_2665477), BV605-conjugated anti-CD19 (RRID:AB_2732057), BV785-conjugated anti-Ly6G (RRID:AB_2740578), Alexa Fluor 700-conjugated anti-Siglec-F (RRID:AB_2739097) (BD Biosciences), PE-Dazzle-conjugated anti-CD11c (RRID:AB_2563655), APC-Cy7-conjugated anti-MHC-II (RRID:AB_2069377), PE-conjugated anti-CD64 (RRID:AB_10612740), and PerCP-Cy5.5-conjugated anti-CD11b (RRID:AB_893232) (BioLegend) antibodies at 4 °C for 30 min and were washed. Next, 2% FBS containing PBS-resuspended samples were assessed on a CytoFLEX (Beckman Coulter, RRID:SCR_019627) and analysed using FlowJo software (Tree star, RRID: SCR_008520, Ashland, OR, USA). The detailed information of antibodies and peptide was summarized in Supplementary Information.

### Histopathology

For histopathological analysis, the right frontal lobes of the lungs were preserved in 10% neutral buffered formalin overnight and embedded in paraffin. Then, the lungs were sectioned at 4–5 μm and stained with haematoxylin and eosin (H&E). A qualified pathologist read the slides in a blinded manner. A scoring system that included examination of the lungs for peribronchiolitis, perivasculitis, alveolitis, “granuloma” formation, and the degree of necrosis was used to give a total lung score for the lungs from each mouse. The lesions were assessed as previously described^19^. Briefly, the number of lesions apparent in a section was counted, and the percentage of involved parenchyma was estimated. The following features were assessed individually: peribronchiolitis, perivascular leukocyte infiltration (“perivasculitis”), alveolitis, “granuloma” formation (i.e., granulomatous inflammation), and necrosis on a scale of 0–5 [0, within normal limits (no change); 1, minimal changes; 2, mild changes; 3, moderate changes; 4, marked changes; and 5, very severe changes].

### Quantification of cytokines

The cytokine levels in lung homogenates from Mtb-infected mice were measured using commercial ELISA kits according to the manufacturers’ instructions. ELISA was used to detect TNF-α (RRID:AB_2575080), IFN-γ (RRID:AB_2575066), and IL-10 (RRID:AB_2574998) (Thermo Fisher Scientific) in the lung homogenates.

### Statistical analysis

Unless indicated otherwise, data are presented as means with standard deviations, median and ranges, or median with standard deviation. Distribution of data was determined with the Shapiro–Wilk test. For immunological and CFU analysis, the significance of differences between samples was assessed by one-way ANOVA followed by Tukey’s post hoc for multiple comparisons. For histological analyses, the Kruskal–Wallis test was used. Statistical analysis was performed using GraphPad Prism version 7.00 for Windows (GraphPad Software, RRID:SCR_002798, La Jolla, California, USA, www. graphpad.com). Group comparisons where *p* < 0.05 were considered significantly different.

### Adherence to ARRIVE guidelines

All protocols involving animals were performed according to the ARRIVE guidelines 2.0 (https://arriveguidelines.org/arrive-guidelines), where the essential 10 and recommended set of details were indicated per specific experimental approach.

## Supplementary Information

Supplementary Information.

## Data Availability

The datasets generated and analysed in this study are available from the corresponding author upon reasonable request.
