# Supplementary material for: BCGΔBCG1419c increased memory CD8+ T cell-associated immunogenicity and mitigated pulmonary inflammation compared with BCG in a model of chronic tuberculosis
Source: Sci Rep. 2022 Sep 22;12:15824. doi: 10.1038/s41598-022-20017-w (PMC9499934; doi:10.1038/s41598-022-20017-w)
Supplement: Supplementary file 1 — Supplementary Information. [file 41598_2022_20017_MOESM1_ESM.docx]

**BCGΔBCG1419c increased memory CD8^+^ T cell-associated immunogenicity and mitigated pulmonary inflammation compared with BCG in a model of chronic tuberculosis**

Kee Woong Kwon^a^, Michel de Jesús Aceves-Sánchez^b^, Cristian Alfredo Segura-Cerda^b^, Eunsol Choi^a^, Helle Bielefeldt-Ohmann^c,d^, Sung Jae Shin^a,e*^, Mario A. Flores-Valdez^b*^

^a^Department of Microbiology, Graduate School of Medical Science, Brain Korea 21 Project, Yonsei University College of Medicine, Seoul 03722, South Korea

^b^Biotecnología Médica y Farmacéutica, Centro de Investigación y Asistencia en Tecnología y Diseño del Estado de Jalisco, Guadalajara, Mexico

^c^Australian Infectious Diseases Research Centre, The University of Queensland, Saint Lucia, QLD, Australia

^d^School of Chemistry and Molecular Biosciences, University of Queensland St. Luica Campus, St Lucia, QLD 4072, Australia

^e^Institute for Immunology and Immunological Disease, Yonsei University College of Medicine, Seoul 03722, South Korea

* Correspondence:

Sung Jae Shin, Department of Microbiology, Yonsei University College of Medicine, Seoul 03722, Republic of Korea. Phone: 82-2-2228-1813; Email: sjshin@yuhs.ac

Mario A. Flores-Valdez, Biotecnología Médica y Farmacéutica, Centro de Investigación y Asistencia en Tecnología y Diseño del Estado de Jalisco, A.C., Av. Normalistas No. 800, Col. Colinas de la Normal, 44270 Guadalajara, Jalisco, Mexico. Email: floresv@ciatej.mx

**Supplementary figures, figure legends, and Supplementary table**


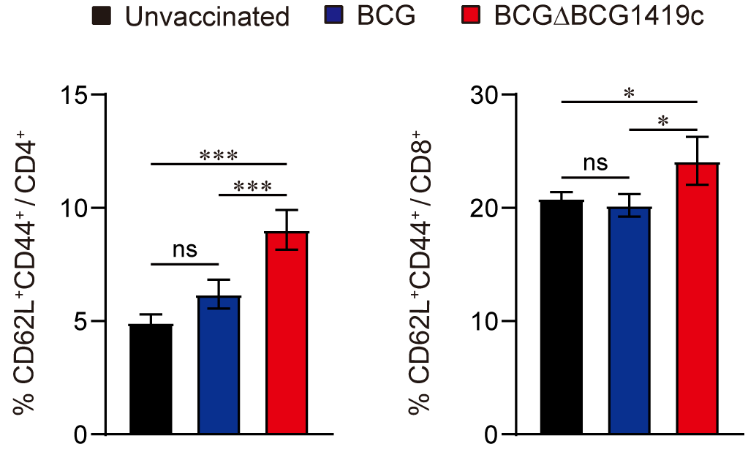


**Fig. S1 Analysis of central memory T cell phenotype in the lung.** Ten weeks after the final vaccination, mice from each group (*n* = 4) were sacrificed. Then the frequencies of CD4^+^CD62L^+^CD44^+^ or CD8^+^CD62L^+^CD44^+^ T-cells were determined by surface staining in the lungs of each vaccinated mouse and presented as bar graphs. The experimental results of one representative experiment are presented as the mean ± SD from 4 mice from each group. One-way ANOVA with post hoc Tukey’s multiple comparison test was used to evaluate the significance. **p* < 0.05 and ****p* < 0.001.

**
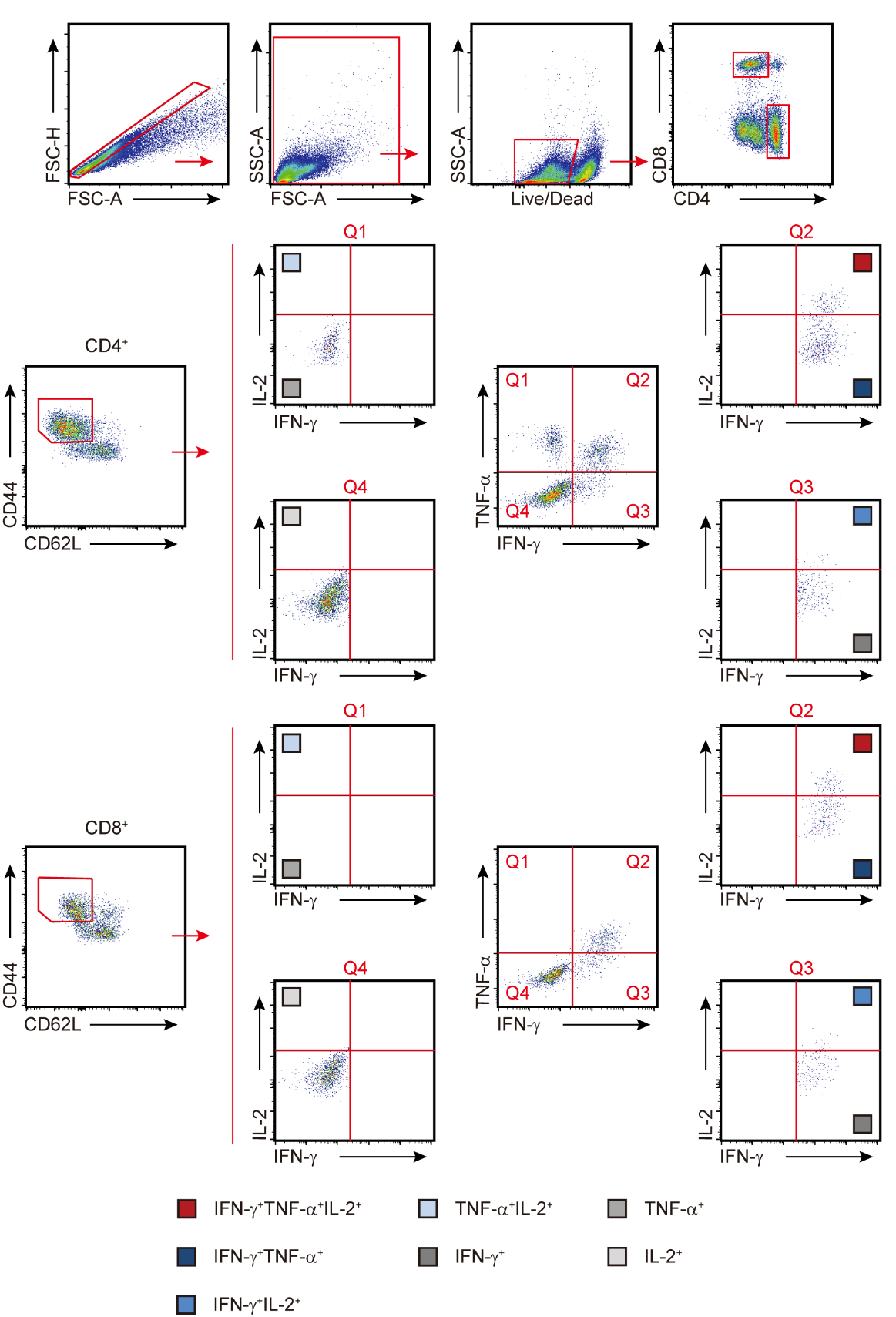
**

**Fig. S2 Flow cytometry gating strategy for dissecting antigen-specific polyfunctional T cells.** All samples stained for surface and intracellular cytokines were gated based on forward scatter (FSC) and side scatter (SSC). T cells were gated based on CD4 and CD8 expression. Specific staining for intracellular cytokines is displayed using CD4^+^CD62L^-^CD44^+^- or CD8^+^CD62L^-^CD44^+^- T cell gating.


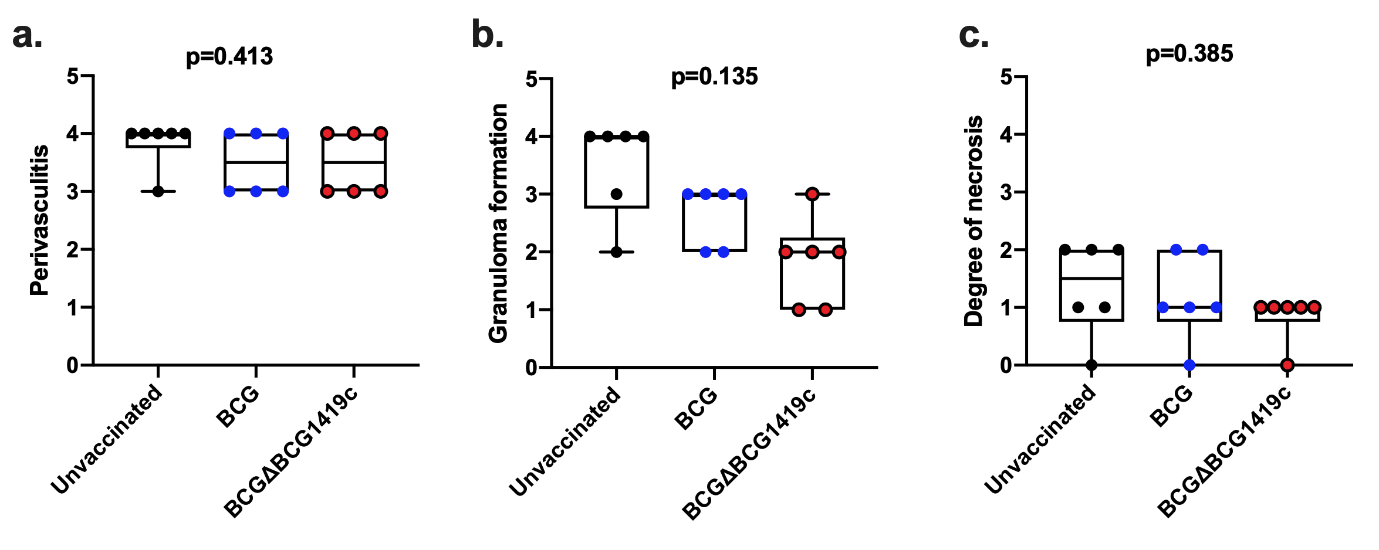


**Fig. S3 Assessment of pathological parameters.** Lungs from vaccinated or unvaccinated mice (*n* = 6/group) were scored for (a) perivasculitis, (b) granuloma formation, and (c) degree of necrosis, at 10 weeks post-infection. Data (*n* = 6) from one representative experiment are presented as a box and whisker plot showing all points. Kruskal-Wallis followed by Dunn’s multiple comparison test was used to evaluate the significance.


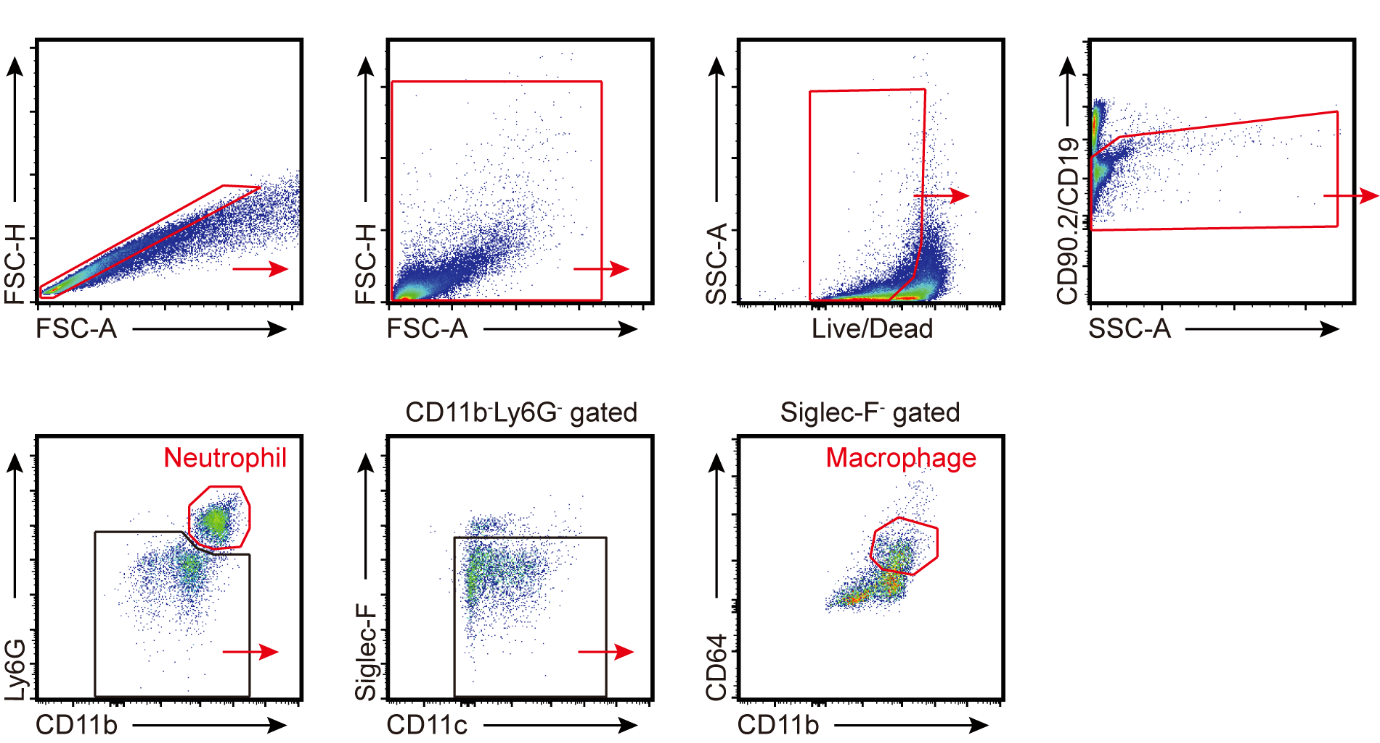


**Fig. S4 Flow cytometry gating strategy for dissecting neutrophils and macrophages in the lung.** Gating strategy for the examination of neutrophils and macrophages in the lung at 10 weeks post-infection.

Supplementary Table S1. List of fluorescently conjugated antibodies and peptides used in this study

| **Antibodies** | **Dilution** | **Source** | **Identifier** |
| --- | --- | --- | --- |
| LIVE/DEAD^TM^ Fixable Viability Dye eFluor^TM^ 780 | 1:1000 | ThermoFisher Scientific | Cat. No. 65-0865 |
| PerCP-Cy5.5-conjugated anti-mouse CD4 (clone RM4-5) | 1:300 | BD Biosciences | Cat. No. 550954  RRID:AB_393977 |
| Brilliant Violet (BV) 786-conjugated anti-mouse CD8a (clone 53-6.7) | 1:300 | BD Biosciences | Cat. No. 563332  RRID:AB_2721167 |
| Violet 450-conjugated anti-mouse CD44 (clone IM7) | 1:300 | BD Biosciences | Cat. No. 560451 RRID:AB_1645273 |
| Alexa Fluor 700-conjugated anti-mouse CD62L (clone MEL-14) | 1:300 | BD Biosciences | Cat. No. 560517 RRID:AB_1645210 |
| BV605-conjugated anti-mouse CD90.2 (clone 53-2.1) | 1:300 | BD Biosciences | Cat. No. 563008 RRID:AB_2665477 |
| BV605-conjugated anti-mouse CD19 (clone 1D3) | 1:300 | BD Biosciences | Cat. No. 563148 RRID:AB_2732057 |
| BV786-conjugated anti-mouse Ly6G (clone 1A8) | 1:300 | BD Biosciences | Cat. No. 740953 RRID:AB_2740578 |
| Alexa Fluor 700-conjugated anti-mouse Siglec-F (clone E50-2440) | 1:300 | BD Biosciences | Cat. No. 565183 RRID:AB_2739097 |
| Unconjugated anti-mouse CD16/32 (clone 93) | 1:400 | BioLegend | Cat. No. 101320 RRID:AB_1574975 |
| PE-conjugated anti-mouse IFN-γ (clone XMG1.2) | 1:200 | BioLegend | Cat. No. 505808 RRID:AB_315402 |
| APC-conjugated anti-mouse TNF-α (clone MP6-XT22) | 1:200 | BioLegend | Cat. No. 506308 RRID:AB_315429 |
| PE-Cy7-conjugated anti-IL-2 (clone JES6-5H4) | 1:200 | BioLegend | Cat. No. 503832 RRID:AB_2561750 |
| PE/Dazzle^TM^ 594-conjugated anti-mouse CD11c (clone N418) | 1:300 | BioLegend | Cat. No. 117348 RRID:AB_2563655 |
| APC-Cy7-conjugated anti-mouse MHC Class II (clone M5/114.15.2) | 1:300 | BioLegend | Cat. No. 107628 RRID:AB_2069377 |
| PE-conjugated anti-CD64 (clone X54-5/7.1) | 1:300 | BioLegend | Cat. No. 139304 RRID:AB_10612740 |
| PerCP-Cy5.5-conjugated anti-CD11b (clone M1/70) | 1:300 | BioLegend | Cat. No. 101228 RRID:AB_893232 |
| **Peptide** | **Sequence** | **Source** | |
| TB10.4_4-12_ | IMYNYPAML | Peptron (Daejeon, South Korea) | |
